# Supplementary material for: Estimating the human mutation rate from autozygous segments reveals population differences in human mutational processes
Source: Nat Commun. 2017 Aug 21;8:303. doi: 10.1038/s41467-017-00323-y (PMC5566399; doi:10.1038/s41467-017-00323-y)
Supplement: Supplementary file 1 — Supplementary Information [file 41467_2017_323_MOESM1_ESM.pdf]

File Name: Supplementary Information
Description: Supplementary Figures, Supplementary Tables and Supplementary Notes.

File Name: Supplementary Data 1
Description: De novo mutations seen in autozygous sequences, PJL Complete Genomics Trios and Scottish Family Health Study, along with their comparisons with the ExAC consortium datasets and partition into mutational spectra.

File Name: Peer Review File
Description:

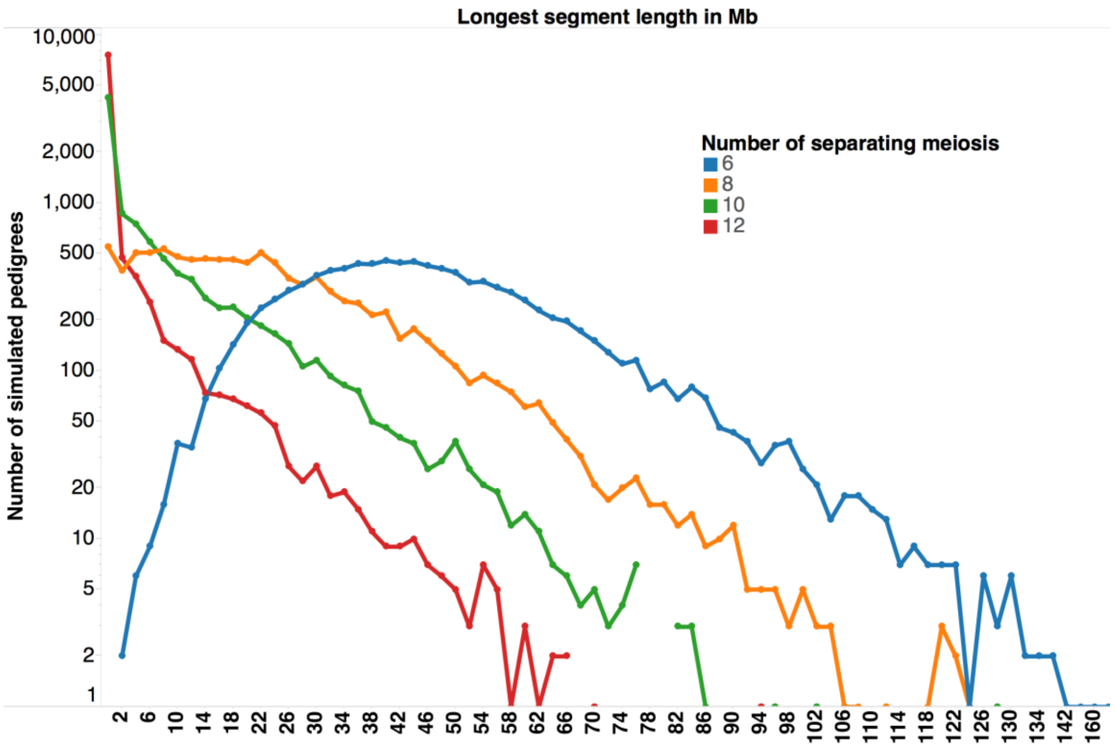

Supplementary Figure 1. Simulated data of showing histograms of the number of pedigrees for which the longest autozygous segment found is of a certain length. Beyond a separation of 10 meioses to the tMRCA, there are fewer than 8% of pedigrees that have an autozygous segment of at least 10Mb.

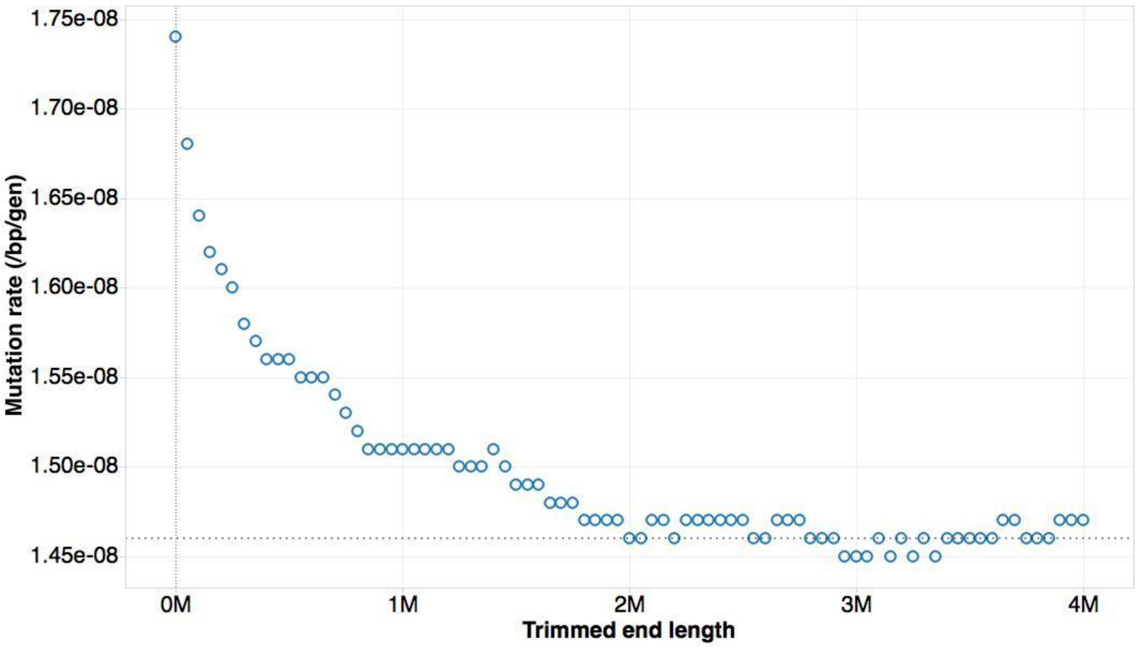

Supplementary Figure 2. The mutation rate estimated from autozygous segments at least 10Mb long that have been further trimmed from each end at a distance given on the x-axis. We see that there is minimal change to the mutation rate estimate beyond 2Mbs of trimming.

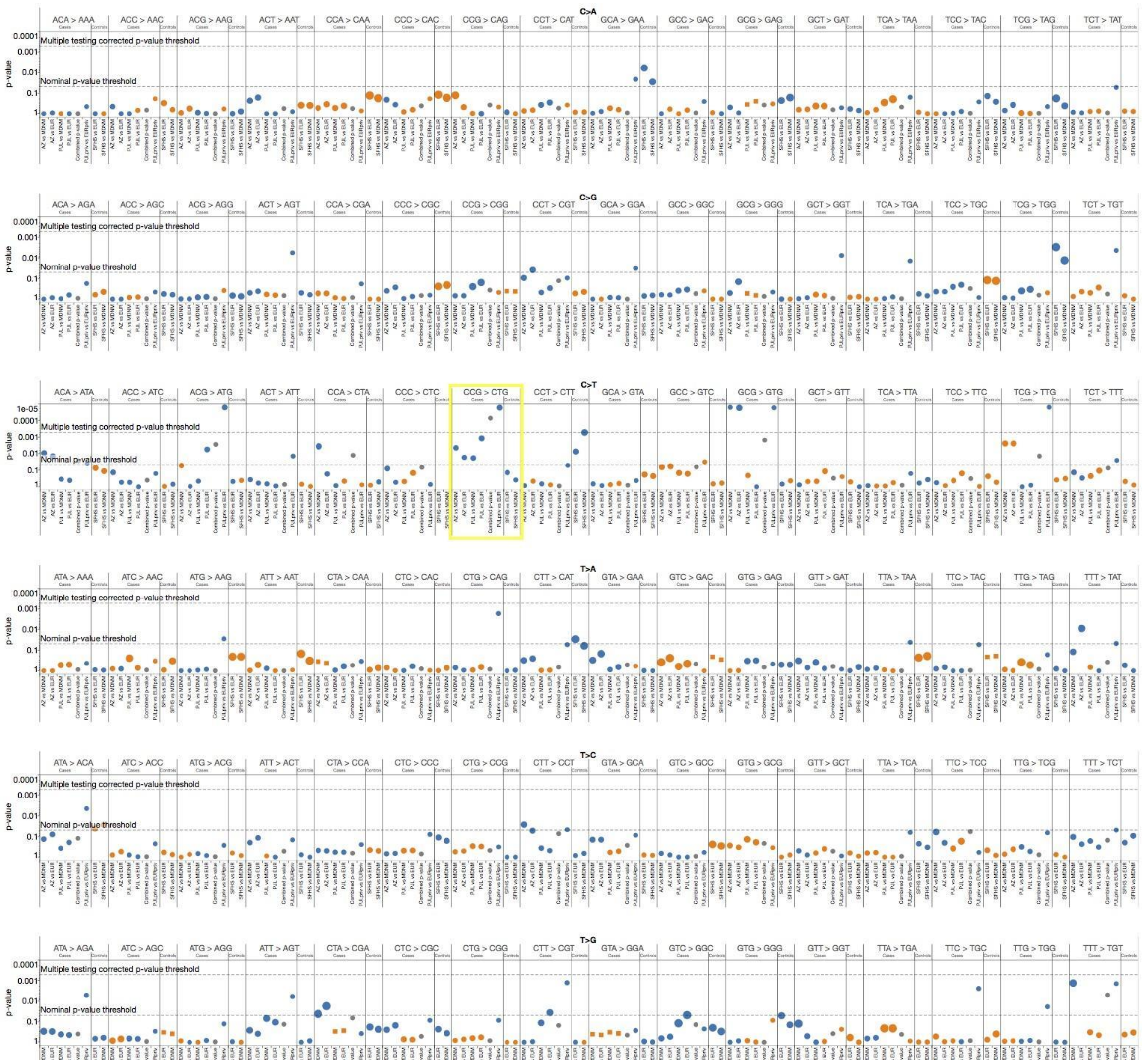

Supplementary Figure 3. Comparisons of the proportion of each of the 96 tri-nucleotide signatures across datasets. Differences in context-specific mutation rate. y-axis: significance of the difference in proportion of DNMs for each signature between 1152 mutations from the autozygosity dataset (AZ) and 850 DNMs from the Complete Genomics trio dataset (PJL) in comparison with 6948 mutations from the meta-analysis dataset (MDNM) and mutations private to Europeans in the 1000 Genomes Project (EURpriv). Additional comparisons for mutations private to the PJL population from the 1000 Genomes Project (PJLpriv) and private to Europeans (EURpriv) shown in rightmost panel. As controls significance of the difference in 747 DNMs from the Scottish Family Health Study (SFHS); Colors (Orange, first population has a lower proportion, Blue, otherwise) and size reflect the sign and fold difference of the test. Comparisons for which de novo mutations have 0 counts shown in squares. The only tri-nucleotide context, 5’ CCG → CTG 3’ that shows experiment wide significance, and consistent direction of effect shown in yellow box.

a

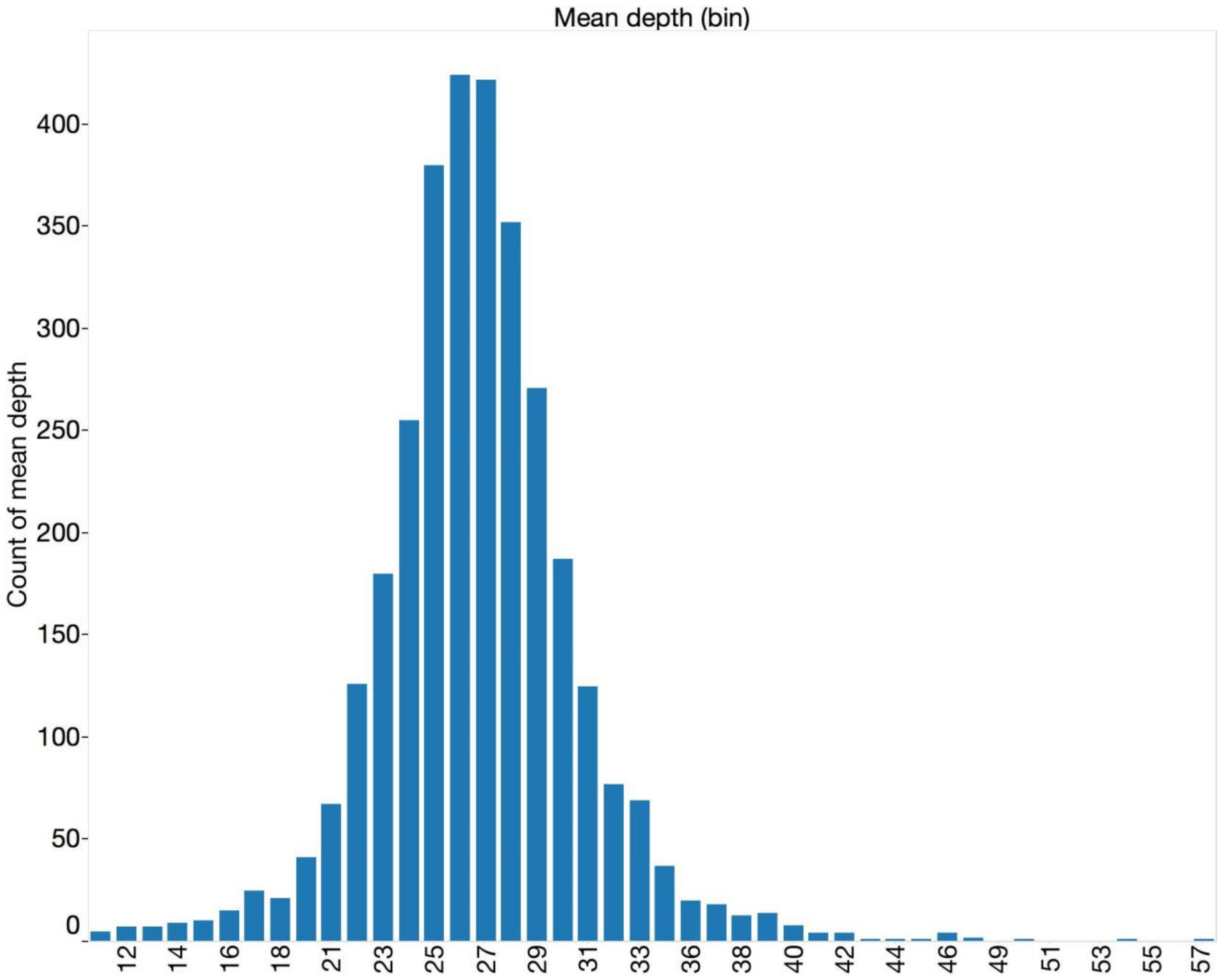
Mean depth (bin)
Count of mean depth
0
50
100
150
200
250
300
350
400
12
14
16
18
21
23
25
27
29
31
33
36
38
40
42
44
46
49
51
53
55
57

b

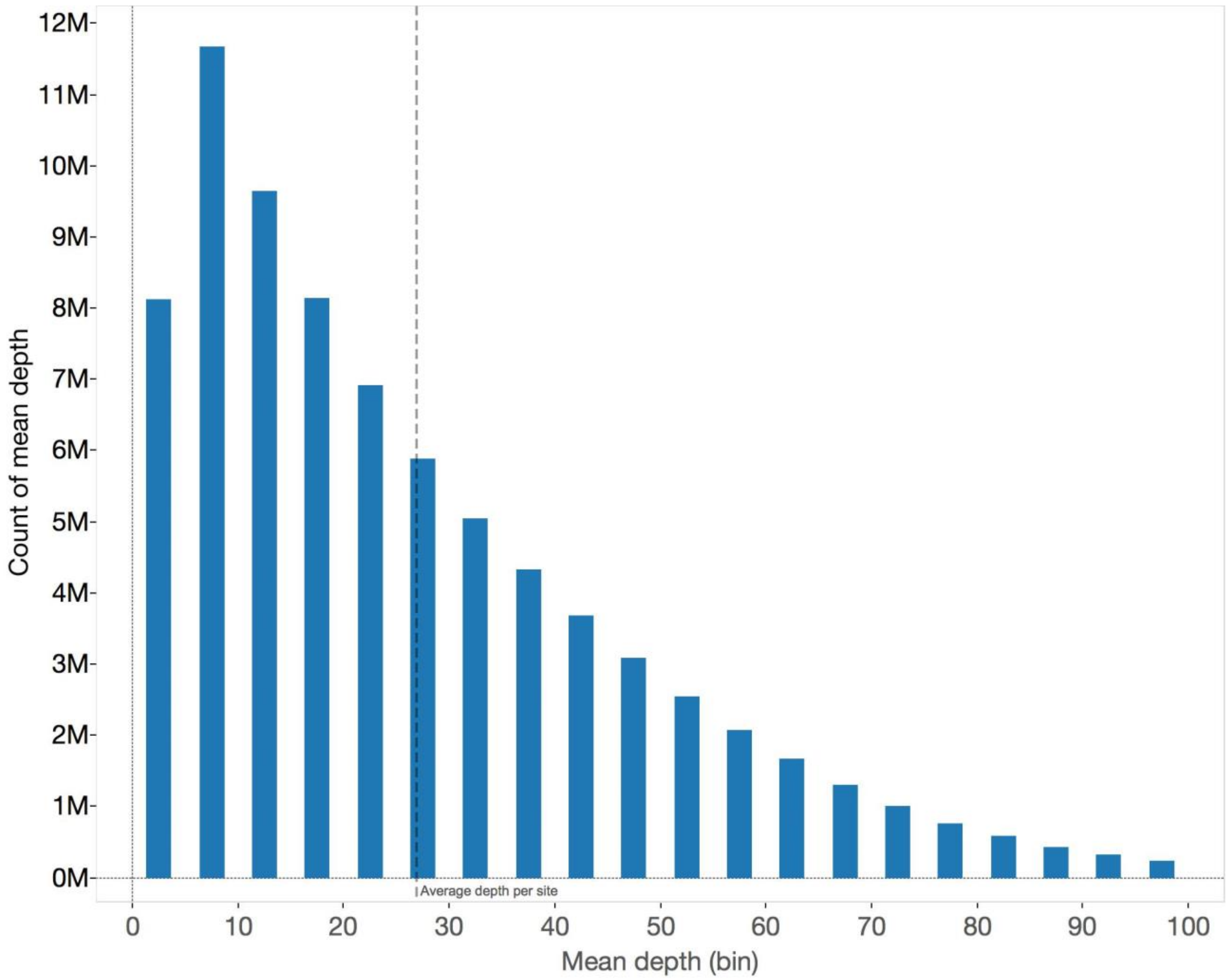

Supplementary Figure 4. Mean depth across sites and individuals on the evaluated portion of the genome

a Histogram of the distribution of mean depth per individual across all sites. **b** Histogram of the mean depth per site across all individuals.

		**Self stated parental relatedness**					
		**First cousin**	**First cousin once removed**	**Second cousin**	**Other blood**	**Other marriage**	**Do not know**
**Inferred Meiosis**	**6 (First cousin)**	835	7	33	29	2	528
	**8 (Second cousin)**	423	1	47	63	15	621
	**10 (Third cousin)**	78	1	13	17	11	356
	**>10 (Not considered**	19	0	6	14	0	103

Supplementary Table 1. Most probable number of separating meioses giving rise to autozygous segment lengths as compared with those from self-stated parental relatedness.

		**Pedigree ascertained relatedness**				
		**Double First cousin**	**First cousin**	**First cousin once removed**	**Second cousin**	**Third cousin**
**Inferred Meiosis**	**6 (First cousin)**	2	46	2	0	0
	**8 (Second cousin)**	0	2	0	5	0
	**10 (Third cousin)**	0	0	0	0	1

Supplementary Table 2. Most probable number of separating meioses giving rise to autozygous segment lengths as compared with those from well studied pedigrees.

Allele Frequency	Percentage of sites identified correctly	Percentage of genotypes identified correctly	False negative rate	False positive rate
**Singleton**	83.77	NA	16.23	3.60
**10%**	94.55	97.22	8.23	1.05
**20%**	94.27	98.10	7.63	0.86
**30%**	93.91	98.17	7.92	0.94

Supplementary Table 3. Estimates of the false negative rates on the allele frequency based on our approach of altering reads to contain a new mutation then remapping them and recalling. Two components to the false negative rate are measured: first the percentage of introduced sites that failed to be called, and second the fraction of introduced heterozygous genotypes that failed to be called at a site that was already known to be polymorphic based on other individuals. The total false negative rate is reflected by aggregating both of these types of error.

Class of mutation	PJL	MDNM
**5’ CCG 3’ → 5’CTG 3’**	54	310
**not 5’ CCG 3’ → 5’ CTG 3’**	795	6592

Supplementary Table 4. 2x2 table showing the number of mutations of the particular class, 5’ CCG 3’ → 5’ CTG 3’ in the PJL complete genomics trios and those from a set of meta de novo mutations ascertained in Europeans.

Dataset	Total Number of DNMs	Type of sequencing	Ancestry
Autozygosity, this dataset (AZ)	1152	28x WES illumina HiSeq 2500	Pakistani
Scottish Family Health Study (SFHS)[6]	747	30x WGS illumina HiSeq 2500	European
Meta de novo mutations[6]	6902	Variable coverage WGS	European
PJL Complete Genomics Trios[31]	850	80x Complete genomics	Pakistani
Mutations private to Europeans in the 1000 Genomes Project excluding singletons[12]	2452300	7.4x WGS illumina HiSeq 2500	European
Mutations private to PJL in the 1000 Genomes Project excluding singletons[12]	162855	7.4x WGS illumina HiSeq 2500	Pakistani

Supplementary Table 5. Table showing a listing of various datasets their acronyms, the total number of DNMs seen and the sequencing technology used along with their ancestry.

Supplementary Note 1

Equation 1:

$$P(het\ in\ dup\ 2 \mid het\ in\ dup\ 1, \alpha, \beta, f) = \frac{P(het\ in\ dup\ 2, het\ in\ dup\ 1 \mid \alpha, \beta, f)}{P(het\ in\ dup\ 1 \mid \alpha, \beta, f)}$$

Equation 2:

$$= \frac{P(het\ in\ dup\ 1, het\ in\ dup\ 2 \mid reality\ is\ \mathrm{hom}\, alt, \alpha, \beta, f) P(reality\ is\ \mathrm{hom}\, alt) + P(het\ in\ dup\ 1, het\ in\ dup\ 2 \mid reality\ is\ het, \alpha, \beta, f) P(reality\ is\ het) + P(het\ in\ dup\ 1, het\ in\ dup\ 2 \mid reality\ is\ ref, \alpha, \beta, f) P(reality\ is\ \mathrm{hom}\, ref)}{P(het\ in\ dup\ 1 \mid reality\ is\ \mathrm{hom}\, alt, \alpha, \beta, f) P(reality\ is\ \mathrm{hom}\, alt) + P(het\ in\ dup\ 1 \mid reality\ is\ het, \alpha, \beta, f) P(reality\ is\ het) + P(het\ in\ dup\ 1 \mid reality\ is\ ref, \alpha, \beta, f) P(reality\ is\ \mathrm{hom}\, ref)}$$

$$= \frac{2f^2(1-\beta)^2 + 2f(1-f)(1-\alpha)^2(1-\beta)^2 + 2f(1-f)\alpha^2\beta^2 + 4f(1-f)(1-\alpha)(1-\beta)(\alpha\beta)}{2f^2(1-\beta) + 2f(1-f)(1-\alpha)(1-\beta) + 2f(1-f)(\alpha\beta) + 2(1-f)^2\alpha}$$
